# Study of Obstacle-Crossing and Pitch Control Characteristic of a Novel Jumping Robot

**DOI:** 10.3390/s21072432

**Published:** 2021-04-01

**Authors:** Jixue Mo, Ze Yan, Bing Li, Fengfeng Xi, Yao Li

**Affiliations:** 1 State Key Laboratory of Robotics and System, Harbin Institute of Technology, Harbin 150001, China; 19s053008@stu.hit.edu.cn (J.M.); libing.sgs@hit.edu.cn (B.L.); 2 School of Mechanical Engineering and Automation, Harbin Institute of Technology, Shenzhen 518052, China; 20s053029@stu.hit.edu.cn (Z.Y.); fengxi@ryerson.ca (F.X.)

**Keywords:** jumping robot, intelligent optimization algorithm, pitch control

## Abstract

In this study, we demonstrated a novel jumping robot that has the ability of accurate obstacle-crossing jumping and aerial pitch control. The novel robot can quickly leap high into the air with a powerful water jet thruster. The robot was designed to overcome multiple general obstacles via accurate jumping. Then a modified whale optimization algorithm (MWOA) was proposed to determine an optimized jumping trajectory according to the form of obstacles. By comparing with classical intelligent optimization algorithms, the MWOA revealed superiority in convergence rate and precision. Besides, the dynamics model of aerial pitch control was built and its effect was verified by the pitch control experiment. Lastly, the robot’s obstacle-crossing experiments were performed and the results validated the robot’s good ability of obstacle-crossing and aerial body righting. We believe the optimization of trajectory and the pitch control are of great help for the jumping robot’s complex jumping and obstacle-crossing performance.

## 1. Introduction

In nature, animals use various locomotion methods to travel across different environments, so that their energy consumptions can be effectively minimized [1]. For instance, the frogs usually apply jumping motion to prey or escape from predators, but sometimes they also crawl in a relatively leisurely situation. Thus, the animal’s multiple locomotion abilities can motivate the innovative design of structure and locomotion mechanism of a multi-locomotion robot. 

The combination of jumping movement and wheeled movement is common in multi-locomotion mode robot, the typical robots include the UMN Scout robot [2], SANDIA wheeled hopping vehicle [3,4], the Boston Dynamics’ sand flea robot [5], etc. The jumping movement enables the robot to overcome large-size obstacles and the wheeled movement makes the robot advance rapidly with low energy consumption. Thus, the wheeled jumping robot has better locomotion ability and obstacle-crossing ability compared with other robots. 

Inspired by the terrestrial and marine animals, researchers have developed various mobile robots excelling at terrestrial jumping [6,7,8,9] or aquatic jumping [10,11,12,13]. Most terrestrial jumping robots rely on the surficial reactive force to realize jumping actions while the aquatic jumping robots usually accomplish aquatic jumping in virtue of the surface tension of water or the reactive force from the water jetting process. The gas-powered water jet propulsion scheme proposed in [12] is a proper way to achieve immediate takeoff and effective aquatic jumping on account of its high power density [14]. A well-designed water jet thruster is able to convert the internal high-pressure gas into its own kinetic energy, and the generated thrust is continuous in both terrestrial and aquatic environments since it depends on the surficial reactive force. In addition, the propellant water is environmentally-friendly and easily obtainable. Therefore, the gas-powered water jet propulsion scheme is applicable for terrestrial jumping robots and aquatic jumping robots. The combination of flexible wheels and powerful water jet thrusters on a robot will definitely improve its terrestrial mobility and aerial payload, which is a significative area pending exploration.

Besides wheeled movement and jumping movement, the ability of aerial attitude control (including pitch control and roll control) is also quite important for the jumping robot. The rapid and effective aerial attitude control can increase the jumping robot’s possibility of a safe landing. Some small animals, such as lizards [15,16,17], are good at controlling their body’s attitude by swinging tails. Inspired by that, several robot prototypes were designed to research the tail’s function in body attitude control. Johnson et al. achieved a legged robot’s pitch angle control and safe landing by swaying its tail [18]. Chang-siu also experimented with wheeled robot’s tail swinging and pitch control [19]. Kohut et al. researched the tail’s effect on dynamic turning characteristics for a miniature legged robot [20].

We have designed a series of water jet thrusters which had a good load capacity and was capable of aquatic jumping and terrestrial jumping [21,22]. However, the water jet thruster can neither achieve aerial attitude control nor accurate trajectory control. Therefore, a novel jumping robot was devised with the proposed water jet thruster in [22], which is the first-ever robotic integration of a wheeled system and a water jet thruster. Moreover, a customized optimization algorithm and aerial pitch control model were proposed to optimize the jumping trajectory and help aerial body righting, respectively. We believe the optimization of trajectory and the pitch control are of great help for the jumping robot’s complex jumping and obstacle-crossing performance. Specifically, the decision criteria for overcoming general obstacles was firstly proposed to generate the alternative jumping trajectory set. Then a modified whale optimization method aiming at optimizing the jumping trajectory was put forward, and its superiority was verified by comparing it with other different optimization algorithms. Afterwards, the dynamics model of aerial pitch control was built and its reasonability was verified by the jumping robot’s free-falling experiment. Lastly, the jumping robot’s jumping and obstacle-crossing experiments were carried out, and the experimental results showed that the proposed robot had the good ability of obstacle-crossing and aerial body righting.

## 2. Methods

### 2.1. Structural Design of the Jumping Robot

**Figure 1:**
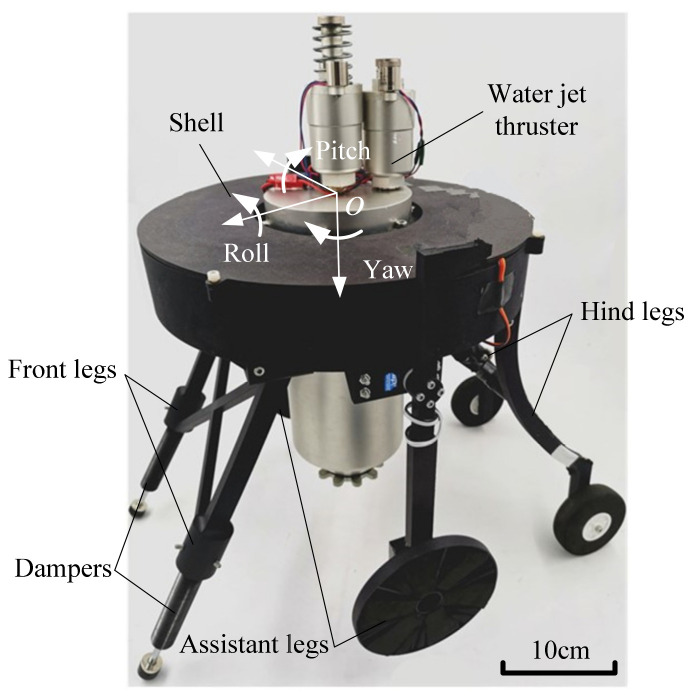
The prototype of the proposed jumping robot.

The prototype of the proposed jumping robot is shown in Figure 1, and its boundary dimension is 487 × 493 × 452 mm^3^. It mainly comprises the water jet thruster, shell, front legs, hind legs, assistant legs, battery and control electronics. The water jet thruster is used to accomplish terrestrial and aquatic jumping movement for the jumping robot. The front legs and hind legs were made of high-performance nylon which has good toughness. Then the dampers and foam wheels were installed on the front legs and hind legs, respectively. Hence the robot can achieve compliant landing by buffering of its front legs and hind legs. In addition, the driving wheels of the assistant legs and the driven wheels of the hind legs enables the jumping robot to execute sustainable wheeled movement on general road. Another function of the assistant legs is serving as the aerial attitude control device, which aiming at increasing the jumping robot’s possibility of safe landing. It is noteworthy that only the robot’s pitch angle can be controlled as the assistant legs have just one rotational degree of freedom. Therefore, the details of the jumping robot’s components are listed in Table 1.

**Figure 2:**
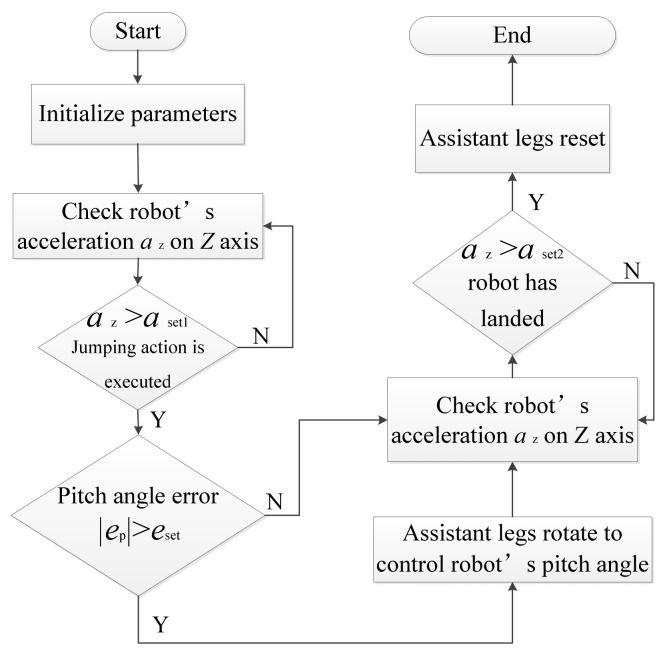
The working process of jumping robot’s control system. *a*_set1_ and *a*_set2_ are two set values of acceleration. *e*_p_ and *e*_set_ are the measured value and set value of pitch angle error, respectively, and 0° is the expected value of pitch angle.

The jumping robot’s control system consists of the control electronics and upper computer software. It is noteworthy that the microcontroller Arduino UNO R3 was applied as the lower computer, and the upper computer can send commands to the upper computer via wireless communication. The jumping robot used 6 channels of PWM signal on the Arduino UNO R3 to control 2 electromagnets (the electromagnets can activate the robot’s jumping action and they are connected with electronic switches which also controlled by PWM signals), 2 servos of assistant legs and 2 servos of driving wheel, respectively. Then the attitude sensor MPU6050 module was used to measure the robot’s accelerations and attitude angles on 3 axes. In addition, the Bluetooth module was applied to achieve the communication between Arduino and upper computer. Lastly, the working process of the jumping robot’s control system are shown in Figure 2.

### 2.2. Decision Criteria of Overcoming General Obstacles

When the jumping robot jumps in the air, the pressure energy of high-pressure gas is converted into kinetic energy of the robot and the propellant water [22]. Supposing the bottle plug of the water jet thruster can be released anytime during the pressurization process, and the duration of the water jetting process is quite short, then the maximum pressure of pressurization *P*_max_ determines the take-off velocity *v*_0_, and the jumping trajectory can be treated as parabola.

**Figure 3:**
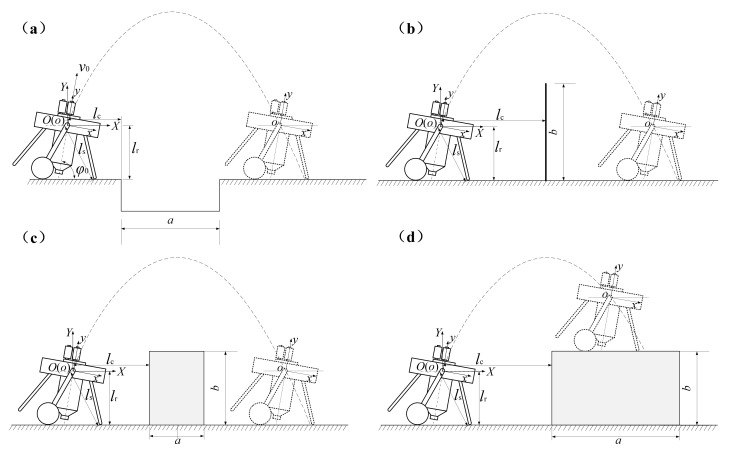
The schematic diagram of jumping robot’s overcoming different obstacles. (**a**) Jumping robot jumps over ditch. (**b**) Jumping robot jumps over wall. (**c**) Jumping robot jumps over platform. (**d**) Jumping robot jumps onto platform.

As shown in Figure 3, taking the robot’s gravity center *o* as the original point and the central axis of water jet thruster as the *y* axis, the carrier coordinate system *xoy* can be established; taking the initial location of robot’s gravity center *O* as the original point and the vertical direction as *Y* axis, the ground coordinate system *XOY* can be established. Then there are two controllable parameters while jumping: the take-off angle *φ*_0_ and the take-off velocity *v*_0_, which are shown in Figure 3a.

It is obvious that different values of *φ*_0_ and *v*_0_ can generate different parabolic trajectory. Supposing the parabolic peak’s coordinate in ground coordinate system is (*x*_p_, *y*_p_), then it can be obtained that:

(1) $$\left\{\begin{array}{l} x_{p}=v_{0}\cos\varphi_{0}\sqrt{2y_{p}/g}\\ y_{p}={\left(v_{0}\sin\varphi_{0}\right)}^{2}/2g \end{array}\right..$$

As the parabola goes through original point, the parabolic equation can be written as:

(2) $$y=-\frac{y_{p}}{x_{p}^{2}}{\left(x-x_{p}\right)}^{2}+y_{p}.$$

In actual situation, the jumping robot may mainly come across three types of obstacles: ditch, wall and platform. Therefore, there are four types of obstacle-crossing tasks: jumping over ditch, jumping over wall, jumping over platform and jumping onto platform (Figure 3). It is noteworthy that the ditch and wall are special cases of platform, as the ditch and wall can be treated as platforms with little height and width respectively. Therefore, the discussions below are for overcoming the platform.

Supposing the platform’s width and height as *a* and *b* respectively, the distance between its left side and the origin *O* is *l*_c_, the height of origin *O* from ground is *l*_r_. In consideration of the safety while landing, there should be a safety distance *l*_s_ (Figure 3c). Therefore, the necessary conditions of jumping over the platform are: the coordinates of the platform’s top vertexes should be located below the parabola; the minimum distance between the platform’s top vertexes and the parabola should be no more than the safety distance *l*_s_. Thus, the decision criteria can be written as:

(3) $$\left\{\begin{array}{l} -\frac{y_{p}}{x_{p}^{2}}{\left(l_{c}-x_{p}\right)}^{2}+y_{p}>b-l_{r}\\ -\frac{y_{p}}{x_{p}^{2}}{\left(l_{c}+a-x_{p}\right)}^{2}+y_{p}>b-l_{r}\\ \min(\sqrt{{\left(x-l_{c}\right)}^{2}+{\left(y-b+l_{r}\right)}^{2}})>l_{s}\\ \min(\sqrt{{\left(x-l_{c}-a\right)}^{2}+{\left(y-b+l_{r}\right)}^{2}})>l_{s} \end{array}\right..$$

Then the constraint conditions are:

(4) $$\left\{\begin{array}{l} y=-\frac{y_{p}}{x_{p}^{2}}{\left(x-x_{p}\right)}^{2}+y_{p}\\ 0\leq x\leq \max(2x_{p},l_{c}+l_{s}+a) \end{array}\right..$$

In the case of jumping onto the platform, the necessary condition is relatively simple: the coordinates of the platform’s top left vertex should be located below the parabola; the minimum distance between the platform’s top left vertex and the parabola should be no more than the safety distance *l*_s_. Thus, the decision criteria can be written as:

(5) $$\left\{\begin{array}{l} -\frac{y_{p}}{x_{p}^{2}}{\left(l_{c}-x_{p}\right)}^{2}+y_{p}>b-l_{r}\\ \min(\sqrt{{\left(x-l_{c}\right)}^{2}+{\left(y-b+l_{r}\right)}^{2}})>l_{s} \end{array}\right..$$

Then the constraint conditions are:

(6) $$\left\{\begin{array}{l} y=-\frac{y_{p}}{x_{p}^{2}}{\left(x-x_{p}\right)}^{2}+y_{p}\\ 0\leq x\leq \max(x_{p}+\sqrt{x_{p}^{2}(y_{p}-b)/y_{p}},l_{s}+l_{c}) \end{array}\right..$$

Hence the value ranges of *φ*_0_ and *v*_0_ can be determined according to Equations (3)–(6) in the case of jumping over the platform and jumping onto the platform.

### 2.3. Modified Whale Optimization Algorithm

After determining the value range of the jumping robot’s *φ*_0_ and *v*_0_, there will be an obstacle-crossing trajectory set for a particular obstacle. It is necessary to optimize the jumping trajectory according to certain indicators by proper optimization algorithms. The whale optimization algorithm (WOA) was proposed by Mirjalili and Lewis in 2016 [23]. It has good performance in terms of convergence rate and convergence precision. However, it also has the problem of outputting a local optimal solution when dealing with complex problems. Thus, a modified whale optimization algorithm was proposed and applied in the optimization of the jumping trajectory.

#### 2.3.1. Standard Whale Optimization Algorithm

The whale optimization algorithm simulates the foraging behavior of humpback whales. The algorithm can be divided into three stages: encircling prey stage, bubbling-net attacking stage and searching for prey.

1. Encircling prey stage. Supposing the scale of the whale population is *N*, the dimension of the searching space is *d*, then the whale individual *i* can be expressed as *X_i_* = (*x_i_*^1^, *x_i_*^2^, …, *x_i_^d^*), *i* = 1, 2, …, *N*. The prey’s position corresponds to the global optimal solution of objective function. Therefore, the mathematic model of the encircling prey stage can be expressed as:

(7) $$D=|C\cdot X_{p}(t)-X(t)|,$$

(8) $$X(t+1)=X_{p}(t)-A\cdot D,$$

where ***D*** is the distance vector between the current optimal solution and the searching individual, *t* is the current iterations, ***X*** (*t*) is the position vector of whale individual and ***X***_p_(*t*) is the current optimal solution. The coefficient vector ***A*** and ***C*** can be calculated by

(9) $$A=2a\cdot r_{1}-a,$$

(10) $$C=2\cdot r_{2},$$

where ***r***_1_ and ***r***_2_ are random vectors in [0,1], and *a* linearly decreases from 2 to 0 with *t*’s growth:

(11) $$a(t)=2(1-\frac{t}{t_{\max}}).$$

2. Bubbling-net attacking stage. This stage stimulates the process that the whale individual moves spirally around the current prey to approach it. The mathematical model of the spiral motion is:

(12) $$X(t+1)=D^{\prime }\cdot e^{bl}\cdot \cos(2\pi l)+X_{p}(t)\;(p\;>\;0.5),$$

where ***D***’ = |***X***_p_(*t*) − ***X***(*t*)| is the distance vector between the current optimal solution and the searching individual, *b* is a constant of defining the shape of the logarithmic spiral and *l* is a random number in [−1,1]. In order to synchronize the behaviors of encircling prey and bubbling-net attack, a probability variable *p* in [0,1] is introduced, thus the mathematical model can be written as:

(13) $$X(t+1)=\left\{\begin{array}{l} X_{p}(t)-A\cdot D\;(p\;<\;0.5)\\ D^{\prime }\cdot e^{bl}\cdot \cos(2\pi l)+X_{p}(t)\;(p\;>\;0.5) \end{array}\right..$$

3. Searching for prey stage. The whale individual decides encircling for prey or searching for prey by the value of |***A***|. When |***A***| > 1, the whale cannot clearly determine the prey’s position. Therefore, it is necessary to pick a random individual in place of the prey, that is:

(14) $$D^{\prime \prime }=\left|C\cdot X_{rand}-X\right|,$$

(15) $$X(t+1)=X_{rand}-A\cdot D^{\prime \prime }.$$

#### 2.3.2. Improvement Strategy

1. Tent mapping strategy. The standard WOA generates an initial population by random method, this may lead to its poor diversity. Therefore, a new chaotic mapping method named tent mapping was applied [24]. For a population with scale *N* and dimension *d*, the chaotic sequence *y* = {*y_d_*, *d* = 1, 2, …, *d*}, *y_d_* = {*y_id_*, *i* = 1, 2, …, *N*} can be firstly obtained by tent mapping with the equation

(16) $$y_{i+1,d}=\left\{\begin{array}{l} 2y_{id},\;y_{id}<0.5\\ 2(1-y_{id}),\;y_{id}\geq 0.5 \end{array}\right..$$

Mapping the chaotic sequence to solution space, a population *X* = {*X_i_*, *i* = 1, 2, …, *N*}, *X_i_* = {*X_id_*, *d* = 1, 2, …, *d*} can be obtained, and

(17) $$X_{id}=X_{\min,d}+y_{id}(X_{\max,d}-X_{\min,d}),$$

where *X_max_*_,*d*_ and *X_min_*_,*d*_ are the upper and lower limit of *X_id_* respectively. Then the reverse population *OX* can be obtained according to *X* by

(18) $$OX_{id}=X_{\max,d}+X_{\min,d}-X_{id}.$$

Lastly, merge the population *X* and *OX*, and sort the new population according to the objective function’s fitness, the top *N* individuals can be taken as the initial population.

2. Non-linear convergence factor and self-adaptive weight strategy. The linear decrease of convergence factor *a* in standard WOA cannot coordinate global searching and local exploitation. Therefore, it is better to make the descending speed of *a* decrease with the progress of iterations *t*. The proposed equation of non-linear convergence factor is

(19) $$a=2[1-\cos(\mu \frac{t}{t_{\max}}\pi +\theta )],$$

where *μ =* 0.5 and *θ* =−π/2 are relevant parameters. Besides, the standard WOA does not take the prey’ guiding force diversity for the whale’s position update with the progress of iterations into consideration. Therefore, a self-adaptive weight can be introduced in the position update equation to utilize the current optimal solution effectively. The equation of self-adaptive weight is:

(20) $$\omega (t)=1-\frac{e^{t/t_{\max}}-1}{e-1}.$$

Then Equation (8) and (12) can be modified as follows:

(21) $$X(t+1)=\omega (t)\cdot X_{p}(t)-A\cdot D,$$

(22) $$X(t+1)=D^{\prime }\cdot e^{bl}\cdot \cos(2\pi l)+\omega (t)\cdot X_{p}(t)\;(p>0.5).$$

3. Diversity variation strategy. The population’s diversity will gradually decrease with the iterative process in standard WOA, which may lead to the algorithm’s premature convergence. Therefore, the variation operation for the population should be conducted when the diversity is below the threshold value. Supposing the fitness value of individual *I* is *f_i_*, *f*_max_, *f*_min_ and *f*_mean_ are the maximum, minimum and mean fitness values of the current population respectively. Then the variance of current population is:

(23) $$\sigma^{2}=\frac{1}{N}\sum_{i=1}^{n}{\left(\frac{f_{i}-f_{\mathrm{mean}}}{f_{\max}-f_{\min}}\right)}^{2}.$$

When the variance is below threshold value, the variation operation can be conducted as follows:

(24) $$X(t+1)=r_{3}\cdot [X_{p}(t)-X(t)]+r_{4}\cdot [X^{\prime }(t)-X(t)],$$

where *r*_3_ and *r*_4_ are random numbers in [0,1], and ***X***’(*t*) is a random individual in the population.

**Figure 4:**
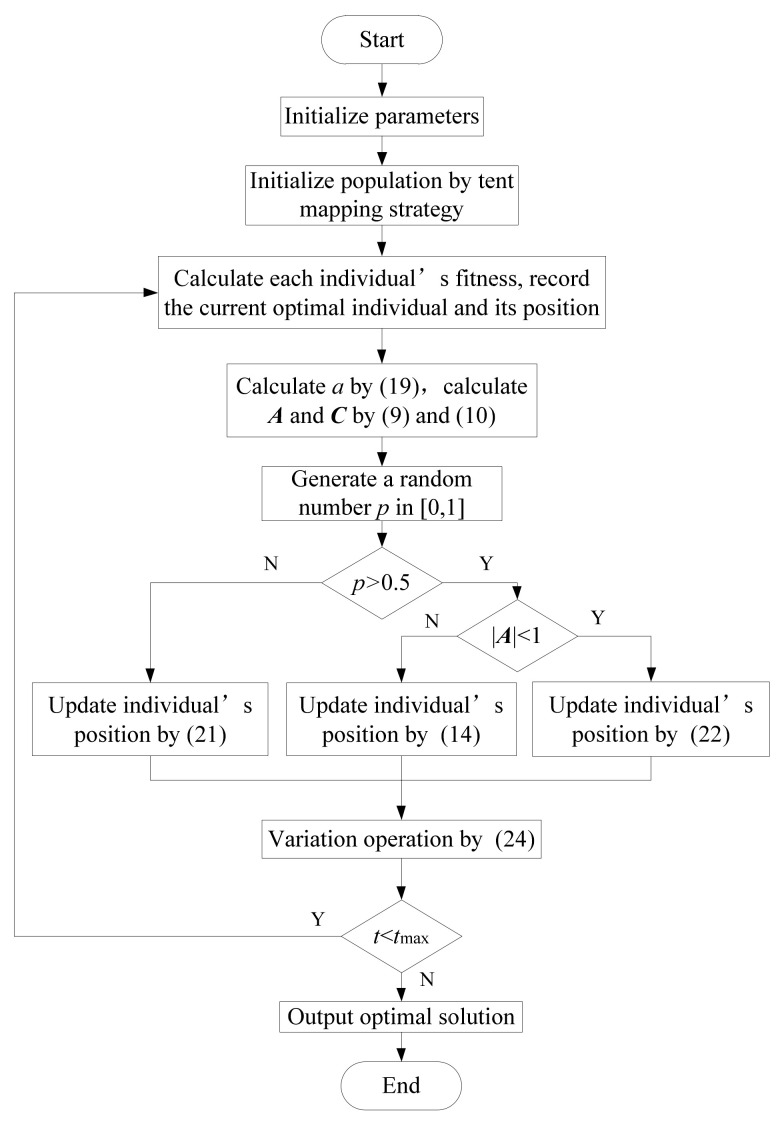
Flow diagram of modified whale optimization algorithm.

Therefore, the flow diagram of MWOA is presented in Figure 4.

#### 2.3.3. Performance Test

In order to test the optimization performance of MWOA, 10 benchmark functions were chosen for the performance test. The details of the benchmark functions are listed in Table A1 (Appendix A). It is noteworthy that *f*_1_~*f*_4_ are unimodal functions, *f*_5_~*f*_8_ are multimodal functions and *f*_9_~*f*_10_ are fixed dimension functions, respectively. The performance test was conducted in two aspects: 1. Test the performance improvement effect of each improvement strategy. 2. Compare the convergence rate and precision of MWOA with standard WOA and another two intelligent optimization algorithms, including DA (Dragonfly Algorithm) [25] and HHO (Harris Hawk Optimization) [26]. The population scale and maximum iterations in both performance tests were 30 and 500 respectively; each benchmark function was tested 30 times, and then the mean value and standard deviation were calculated.

#### 2.3.4. Fitness Function

After determining the specific optimization algorithm, the conditions and objective of jumping trajectory optimization should also be determined. Thus, the condition was: the details of jumping robot’s location, obstacle’s location and size were known; the objective was: to optimize the two take-off parameters (take-off angle *φ*_0_ and take-off velocity *v*_0_) under the fitness function for a specific obstacle-crossing task.

1. Constraint condition of take-off parameters. The jumping trajectory is determined by the take-off parameters *φ*_0_ (rad) and *v*_0_ (m/s), while *v*_0_ was determined by the pressurization pressure, jumping robot’s total mass and the mass of propellant water inside the water jet thruster. The mapping relationship between *v*_0_ and these variables can be obtained by the theoretical jumping model in [22]. Then it is noticeable that the water jet thruster’s propulsive performance would not be so good if *φ*_0_ takes a small value, because the gas–water interface inside the thruster would have a relatively small angle with the thruster’s inner wall and the effect of the water-jetting process would be weaken. Thus, according to the actual situation, the constraint condition of *φ*_0_ and *v*_0_ are given to be:

(25) $$\left\{\begin{array}{l} \pi /3<\varphi_{0}<\pi /2\\ 4<v_{0}<8 \end{array}\right..$$

2. Fitness function. The fitness function comprises three parts: horizontal jumping distance, vertical jumping distance and landing safety. If the robot confronts a ditch or a flat obstacle, the horizontal jumping distance is its priority. Thus, the fitness function of the horizontal jumping ability is

(26) $$f_{o1}=\frac{2v_{0}^{2}\cos\varphi_{0}sin\varphi_{0}/g}{\max(2v_{0}^{2}\cos\varphi_{0}sin\varphi_{0}/g)},$$

where the denominator is the maximum value of the numerator in the constraint condition. If the robot confronts a wall or a slim obstacle, the vertical jumping distance is its priority. Thus, the fitness function of the vertical jumping ability is

(27) $$f_{o2}=\frac{v_{0}^{2}sin^{2}\varphi_{0}}{v_{0\max}^{2}},$$

where *v*_0max_ is the upper limit of *v*_0_. If the robot lands on hard ground with high speed, it can be impacted violently and toppled easily. Thus, the fitness function of landing safety is

(28) $$f_{o3}=1-\frac{{\left(v_{0}^{}-4\right)}^{2}}{32}-\frac{{\left(\varphi_{0}^{}-0.45\pi \right)}^{2}}{2{\left(0.1167\pi \right)}^{2}}.$$

It is noteworthy that these three indicators restrict each other, and they cannot achieve the best value simultaneously. Therefore, each indicator can be weighted and the total fitness function is:

(29) $$F(\varphi_{0},v_{0})=\sum_{i=1}^{3}\omega_{i}f_{oi}(\varphi_{0},v_{0}),$$

where *ω_i_* is the weight coefficient and *ω*_1_ + *ω*_2_ + *ω*_3_ = 1.

### 2.4. Dynamics Model of Aerial Pitch Control

The dynamics model for the jumping robot’s aerial pitch control belongs to the dynamics of coupled rigid bodies while free falling, which were studied by relevant researchers. Yang et al. built the dynamics model of two rigid bodies connected by a spherical joint and then proposed the controllers by input–output linearization [27]. Agrawal et al. applied differential flatness to achieve the control of two coupled rigid bodies [28]. Chang-Siu et al. put forward the non-linear control model for a robot with a two-link tail and two degree-of-freedom actuations [29]. 

**Figure 5:**
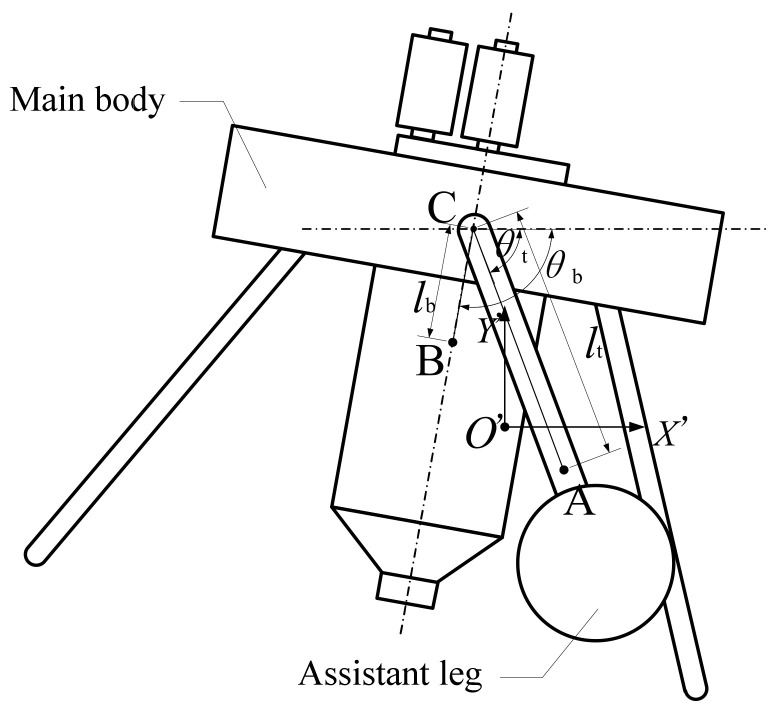
The schematic diagram of jumping robot in the air.

In order to achieve a reliable and effective aerial pitch control for the jumping robot, the dynamics model was first built. For simplification, the robot was seen as being composed of two parts: the main body and the assistant legs; then the robot only rotates in the pitching direction. Since the water jet thruster’s propellant water is drained in a short period of time, the dynamics model assumes that the robotic system does not contain propellant water during the aerial pitch control process. As shown in Figure 5, the assistant leg’s centroid is A, its center of rotation on the main body is C, the main body’s centroid is B and the whole robot’s centroid is *O’*. Then a dynamic coordinate system *O’X’Y’* was built where the *X’* axis and *Y’* axis are along the horizontal direction and the vertical direction, respectively.

Then the robot system’s dynamics model can be derived by the Euler–Lagrange method. Due to the establishment of *O’X’Y’*, the robot’s translational movement can be decoupled from its rotational movement [30]. As the robot’s translational movement is a parabolic movement [31], only the rotational movement is discussed in this section. Therefore, the Lagrangian of the robot system can be written as [32]:

(30) $$\left\{\begin{array}{l} L=\frac{1}{2}(m_{t}v_{A}{}^{2}+I_{t}{\dot{\theta }}_{t}^{2})+\frac{1}{2}(m_{b}v_{B}{}^{2}+I_{b}{\dot{\theta }}_{b}^{2})\\ \;=\frac{1}{2}(m_{t}v_{A}{}^{2}+m_{b}v_{B}{}^{2}+I_{t}{\dot{\theta }}_{t}^{2}+I_{b}{\dot{\theta }}_{b}^{2})\\ v_{A}=\frac{m_{b}}{2m_{t}+m_{b}}({\dot{\theta }}_{t}l_{t}-{\dot{\theta }}_{b}l_{b})\\ v_{B}=\frac{m_{t}}{2m_{t}+m_{b}}({\dot{\theta }}_{t}l_{t}-{\dot{\theta }}_{b}l_{b}) \end{array}\right.\;,$$

where *m*_b_ and *m*_t_ are the mass of main body and assistant legs, respectively; *I*_b_ and *I*_t_ are the rotational inertia of main body and the assistant legs, respectively; *θ*_b_ and *θ*_t_ are the angle of main body and assistant leg relative to *X’* axis, respectively, and *θ*_m_ = *θ*_b_ − *θ*_t_ is the angle of main body relative to assistant leg.

Then applying the Euler–Lagrange method to the robot system, it can be obtained that:

(31) $$\left\{\begin{array}{l} \frac{d\left(\frac{\partial L}{\partial {\dot{\theta }}_{t}}\right)}{dt}-\frac{\partial L}{\partial \theta_{t}}=\tau \\ \frac{d\left(\frac{\partial L}{\partial {\dot{\theta }}_{b}}\right)}{dt}-\frac{\partial L}{\partial \theta_{b}}=-\tau \end{array}\right.\;.$$

It is noteworthy that there is only one external input (the torque *τ* of assistant leg’s servo), so the dynamics Equations can be obtained by substituting (30) into (31):

(32) $$\left\{\begin{array}{l} M{\ddot{\theta }}_{t}-K\cos\theta_{m}{\ddot{\theta }}_{b}+K\sin\theta_{m}{\dot{\theta }}_{b}^{2}=\tau \\ N{\ddot{\theta }}_{b}-K\cos\theta_{m}{\ddot{\theta }}_{t}-K\sin\theta_{m}{\dot{\theta }}_{t}^{2}=-\tau \\ M=2I_{t}+2m_{t}m_{b}/(m_{t}+m_{b})l_{t}{}^{2}\\ N=I_{b}+2m_{t}m_{b}/(m_{t}+m_{b})l_{b}{}^{2}\\ K=2m_{t}m_{b}/(m_{t}+m_{b})l_{b}l_{t} \end{array}\right.\;.$$

For that dynamics equation, there are two controllable parameters, *θ*_b_ and *θ*_t_, while there is only one input parameter, *τ*. However, only *θ*_b_ should be precisely controlled, so the two dynamics equations in (32) can be properly transformed to one which only contains the parameter *θ*_m_ = *θ*_b_ − *θ*_t_. Firstly, the equations of *θ*_b_ and *θ*_t_ can be attained according to (32) as

(33) $$\left\{\begin{array}{l} {\ddot{\theta }}_{b}=\frac{SM{\dot{\theta }}^{2}{}_{t}-Q\tau -SK\cos\theta_{m}{\dot{\theta }}_{b}^{2}}{P}\\ {\ddot{\theta }}_{t}=\frac{-SN{\dot{\theta }}^{2}{}_{b}+R\tau +SK\cos\theta_{m}{\dot{\theta }}_{t}^{2}}{P}\\ P=MN-K^{2}\cos^{2}\theta_{m}\\ S=K\sin\theta_{m}\\ Q=M-K\cos\theta_{m}\\ R=N-K\cos\theta_{m} \end{array}\right.\;.$$

As ${\ddot{\theta }}_{m}={\ddot{\theta }}_{b}-{\ddot{\theta }}_{t}$, thus it can be derived from (33) that

(34) $${\ddot{\theta }}_{m}={\ddot{\theta }}_{b}-{\ddot{\theta }}_{t}=\frac{SQ{\dot{\theta }}^{2}{}_{t}+SR{\dot{\theta }}^{2}{}_{b}-Q\tau -R\tau }{P}.$$

Then the parameters ${\dot{\theta }}_{b}$ and ${\dot{\theta }}_{t}$ can be eliminated by the theorem of angular momentum conservation. Supposing the robotic system’s total angular momentum is zero, then the robotic system’s angular momentum is

(35) $$H_{0}=\frac{\partial L}{\partial {\dot{\theta }}_{t}}+\frac{\partial L}{\partial {\dot{\theta }}_{b}}=(M-Kcos\theta_{m}){\dot{\theta }}_{t}+(N-Kcos\theta_{m}){\dot{\theta }}_{b}=0.$$

Thus, the equations of ${\dot{\theta }}_{b}$ and ${\dot{\theta }}_{t}$ can be derived as

(36) $$\left\{\begin{array}{l} {\dot{\theta }}_{b}=\frac{Q}{R+Q}{\dot{\theta }}_{m}\\ {\dot{\theta }}_{t}=\frac{-R}{R+Q}{\dot{\theta }}_{m} \end{array}\right..$$

Taking the integral of the first Equation of (36), it can be obtained that

(37) $$\theta_{b}(t)=\theta_{b}(0)+\int_{0}^{t}\frac{Q}{\left(R+Q\right)}{\dot{\theta }}^{}{}_{m}dt,$$

where *θ*_b_(0) is the initial value of *θ*_b_ of the aerial pitch control process. Then replacing the integration variable *t* with *θ*_m_, it can be obtained that:

(38) $$\theta_{b}(t)=\int_{\theta_{m0}}^{\theta_{\mathrm{mt}}}\frac{Q}{\left(R+Q\right)}d\theta_{m}+\theta_{b}(0),$$

where *θ*_m0_ and *θ*_mt_ are *θ*_m_’s values at the initial and end moment of the aerial pitch control process respectively. Then combining Equations (32) and (33), the antiderivative of *Q*/(*R* + *Q*) in (38) can be calculated by MATLAB 2016 [33], and the calculation result is

(39) $$\left\{\begin{array}{l} f(\theta_{m})=\frac{\theta_{m}}{2}-\frac{U\cdot V}{W}\\ U=\ln[\frac{\cos\theta_{m}(M+N)-2K+\sin\theta_{m}\sqrt{4K^{2}-{\left(M+N\right)}^{2}}}{M+N-2K\cos\theta_{m}}]\\ V=K(M+N)-2KM\\ W=2K\sqrt{4K^{2}-{\left(M+N\right)}^{2}} \end{array}\right.,$$

Thus, Equation (38) can be transformed to be

(40) $$\theta_{b}(t)-\theta_{b}(0)=f(\theta_{\mathrm{mt}})-f(\theta_{m0}).$$

Therefore, once the initial value *θ*_b_(0), final value *θ*_b_(*t*) of *θ*_b_ and the initial value *θ*_m0_ of *θ*_m_ are determined, the final value *θ*_m*t*_ of *θ*_m_ can be calculated and then the necessary rotation angle of assistant leg Δ*θ*_t_ = *θ*_t_(*t*) − *θ*_t_(0) can also be determined. In most cases, *θ*_b_(*t*) = −π/2, and the ranges of *θ*_b_(0) and *θ*_m0_, were given to be [−*π*,0] and [−*π*/2, *π*/2], respectively.

### 2.5. Aerial Pitch Control Experiments of Jumping Robot

Since the dynamics model of aerial pitch control of the jumping robot were proposed, its correctness and reliability should be verified by experiments. The experimental method lets the robot fall freely from the given initial height and records the pitch angle’s variation with or without the effect of aerial pitch control movement. The experiments were divided into two groups and each group contained five experiments. There was 580 mL propellant water in the water jet thruster’s inner tank in the Group 2, while there was no water in Group 1. Thus, the experimental scheme of aerial pitch control was shown in Table 2. It is noteworthy that there were no aerial pitch control actions in experiment No. 5 and No. 10, which were meant to be the control experiments of experiment No. 4 and No. 9, respectively; besides, the robot’s pitch angle *θ*_p_ = *θ*_b_ + *π*/2. In order to prevent obvious error in the robot’s roll direction during the free falling process, the robot’s initial roll angle was controlled to be zero and its center of gravity in the roll direction was also balanced.

The assistant leg’s theoretical rotation angle of each experiment can be calculated from Equation (40). For the jumping robot, the related parameters in (40) are: *m*_t_ = 0.438 kg, *m*_b_1_ = 3.922 kg, *m*_b_2_ = 4.502 kg, *l*_t_ = 0.146 m, *l*_b_ = 0.041 m, *I*_t_ = 0.00668 kg·m^2^, *I*_b_1_ = 0.085 kg·m^2^ and *I*_b_2_ = 0.087 kg·m^2^. It is noteworthy that *m*_b_1_ and *m*_b_2_ are the main body’s mass in Group 1 and Group 2; *I*_t_ are the total rotational inertia of the robot’s assistant legs as they were meant to rotate simultaneously; *I*_b_1_ and *I*_b_2_ are the main body’s rotational inertia in Group 1 and Group 2. The expected value of the main body and the initial angle of the main body relative to the assistant leg were given to be *θ*_b_(*t*)= −π/2 and *θ*_m0_ = 0, respectively. Hence for each experiment in Group 1 and Group 2, the corresponding assistant leg’s theoretical rotation angle Δ*θ*_t_ = *θ*_t_(*t*) − *θ*_t_(0) can be calculated according to Equation (40), and the calculation results were listed in Table 2.

In addition, the falling and aerial pitch control process of the jumping robot was recorded by a high-speed camera (i-speed 221, iX Cameras, Britain), and the shooting frame rate was 400 Hz. The variation of the robot’s pitch angle *θ*_p_ was obtained from the analysis of experimental video in the motion analysis software ProAnalyst, and the sampling frequency was 400 Hz; the robot’s height from the ground was measured by a telemeter rod.

### 2.6. Jumping Experiments of the Robot

#### 2.6.1. Trajectory Accuracy Verification Experiments

In order to ensure the experimental jumping trajectory’s accuracy, it is necessary to carry out the trajectory accuracy verification experiments, which contained four terrestrial jumping experiments of the jumping robot with different take-off parameters (take-off angle *φ*_0_ and take-off velocity *v*_0_). It is noteworthy that the terrestrial jumping movements were conducted by the water jetting process of the water jet thruster’s inner tank. The take-off velocity *v*_0_ was positively related to the maximum pressurization pressure *P*_max_ when the robot’s total mass and propellant water’s mass were determined, and the mapping relationship between *v*_0_ and *P*_max_ were given by the theoretical jumping model in [22]. Thus, the take-off parameters of four experiments were: a. *φ*_0_ = 87°, *P*_max_ = 1.81 MPa; b. *φ*_0_ = 87°, *P*_max_ = 2.23 MPa; c. *φ*_0_ = 78°, *P*_max_ = 1.81 MPa; d. *φ*_0_ = 78°, *P*_max_ = 2.23 MPa.

In addition, the volumes of propellant water in four experiments were all 580 mL, and the jumping robot’s total mass was 4.36 kg. Thus, the corresponding theoretical take-off velocities with given maximum pressurization pressures of 1.81 MPa and 2.23 MPa can be calculated to be 5.36 m/s and 6.46 m/s, respectively.

In the robot’s terrestrial jumping experiment, the propellant water was firstly injected into the water jet thruster. Then the robot’s initial attitude was adjusted according to the given take-off angle. The robot started jumping movement a while after the liquid nitrogen was injected and the water jet thruster’s inner tank was sealed. The jumping process was recorded by a digital camera (FDR-AX45, Sony, Tokyo) and the experimental jumping trajectory of robot’s centroid was extracted from the experimental video by ProAnalyst.

#### 2.6.2. Obstacle-Crossing Experiments

After the accuracy of robot’s experimental jumping trajectory was verified, the obstacle-crossing experiments can be conducted with the third optimized take-off parameters whose priority was landing safety. These experiments were meant to verify the robot’s abilities of obstacle-crossing and aerial pitch control. Similarly, the volume of propellant water inside the water jet thruster’s inner tank was 580 mL, and the jumping robot’s total mass was 4.36 kg. As the optimized take-off parameters of the experiments of jumping onto the platform and jumping over the platform were both *φ*_0_ = 76.0° and *v*_0_ = 5.57 m/s, then the corresponding maximum pressurization pressure of the water jet thruster was 1.89 MPa.

As shown in Figure 3c,d, the experimental obstacle-crossing parameters were in accordance with those of the optimization process. Thus, for the experiment of jumping onto the platform, *l*_c_ = 0.615 m and *b* = 1.04 m; and for the experiment of jumping over the platform, *l*_c_ = 0.57 m, *a* = 0.105 m and *b* = 0.67 m. The experimental procedure was the same with that of the trajectory accuracy verification experiment. In addition, the robot’s experimental jumping trajectories and pitch angle curves were obtained by analysis of experimental video in ProAnalyst and the onboard attitude sensor respectively.

## 3. Results and Discussion

### 3.1. Modified Whale Optimization Algorithm

#### 3.1.1. Performance Improvement Effect of Each Improvement Strategy

In order to analyze the performance improvement effect of each improvement strategy, the 10 benchmark functions were used to test each improvement strategy. Therefore, the WOA modified by tent mapping strategy was named MWOA-I; the WOA modified by non-linear convergence factor and self-adaptive weight strategy was named MWOA-II; the WOA modified by diversity variation strategy was named MWOA-III. The comparison results were shown in Table 3.

The bold column for each benchmark function in Table 3 is the best result. According to Table 3, the MWOA had best performances in 8 out of the 10 benchmark functions, then the MWOA-II and MWOA-III had 6 best results and 4 best results respectively. Therefore, it can be concluded that the non-linear convergence factor and self-adaptive weight strategy had a larger performance improvement effect than the other two strategies. However, the MWOA, which combined the three strategies still outnumber any other algorithms which utilized only one strategy in the best optimization results.

#### 3.1.2. Performance Comparison with Other Intelligent Optimization Algorithms

**Figure 6:**
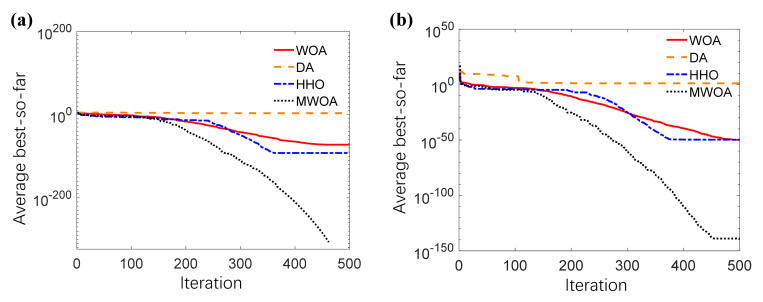
Convergence curves of four intelligent optimization algorithms. (**a**) Convergence curves for *f*_1_. (**b**) Convergence curves for *f*_2_. Average best-so-far means the average of the solution obtained so far in each iteration over 30 runs.

**Figure A1:**
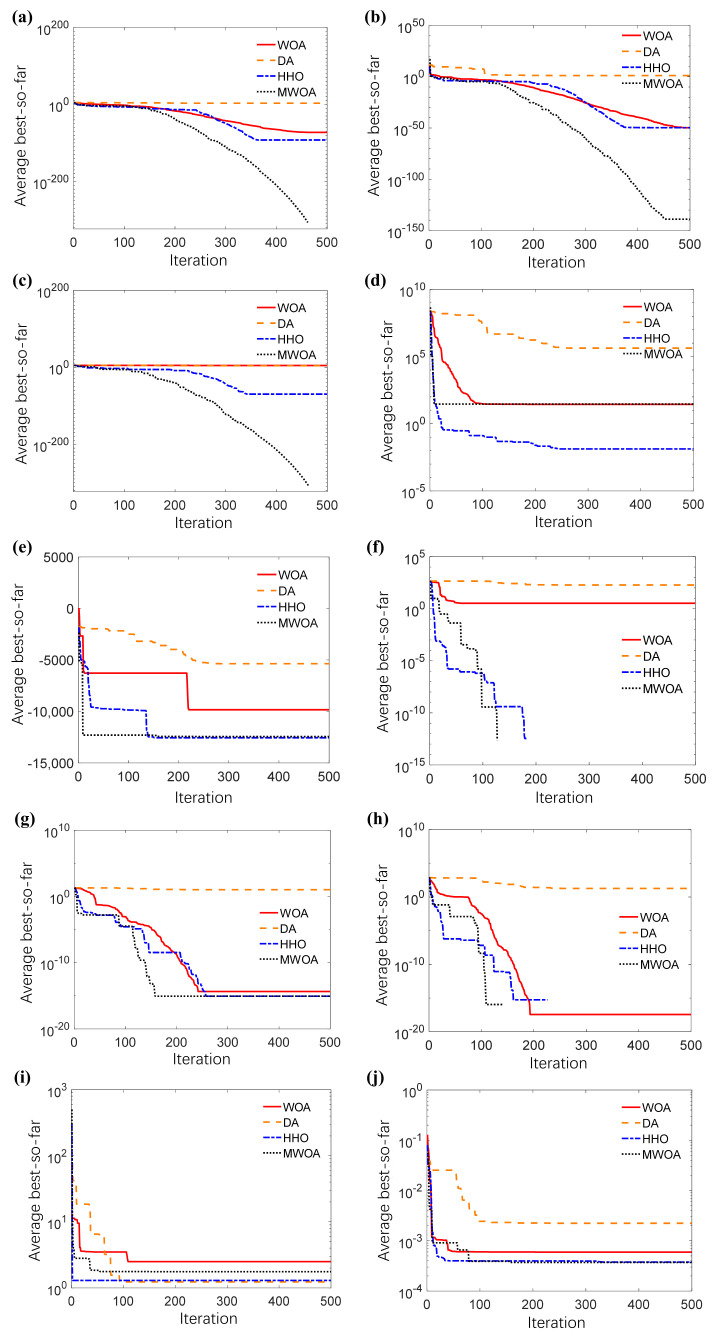
Convergence curves of four intelligent optimization algorithms. (**a**) Convergence curves for *f*_1_. (**b**) Convergence curves for *f*_2_. (**c**) Convergence curves for *f*_3_. (**d**) Convergence curves for *f*_4_. (**e**) Convergence curves for *f*_5_. (**f**) Convergence curves for *f*_6_. (**g**) Convergence curves for *f*_7_. (**h**) Convergence curves for *f*_8_. (**i**) Convergence curves for *f*_9_. (**j**) Convergence curves for *f*_10_.

In order to compare the MWOA’s performance with other intelligent optimization algorithms, the 10 benchmark functions were used again to test the performance of WOA, DA, HHO and MWOA. The comparison results were shown in Table 4, and the convergence curves of these four algorithms for *f*_1_ and *f*_2_ were shown Figure 6 (the other convergence curves were presented in Figure A1).

Similarly, the bold column for each benchmark function in Table 4 is the best result. According to Table 4, the MWOA had the 7 best results among the 10 benchmark functions, then the HHO and DA had the 5 best results and 1 of the best results, respectively. Thus, it can be concluded that the convergence precision and stability of the MWOA were the best according to the evaluation indicators of the mean value and standard deviation.

By analyzing the convergence curves of four intelligent optimization algorithms, it can be concluded that the MWOA had the fastest convergence speeds in 6 benchmark functions (*f*_1_, *f*_2_, *f*_3_, *f*_6_, *f*_7_ and *f*_8_), which surpassed the other algorithms. Compared with standard WOA, the MWOA had better convergence speeds in all 10 benchmark functions. For the optimization of *f*_6_, *f*_7_, *f*_8_ and *f*_10_, the convergence curves of MWOA showed multiple inflection points while descending, which means its ability of escaping from local optimal solution was obviously improved. Thus, by performance comparison with other intelligent optimization algorithms, the reasonability and effectiveness of MWOA can be proved.

#### 3.1.3. Optimization of Jumping Trajectory

After determining the total fitness function, the centroid jumping trajectory of jumping onto a platform and jumping over a platform can be optimized under different weight coefficient combinations. As shown in Figure 3c,d, the relevant parameter values of jumping onto the platform are: *l*_c_ = 0.615 m, *l*_s_ = 0.32 m, *l*_r_ = 0.30 m and *b* = 1.04 m; then the relevant parameter values of jumping over platform are: *l*_c_ = 0.57 m, *l*_s_ = 0.32 m, *l*_r_ = 0.30 m, *a* = 0.105 m and *b* = 0.67 m. It is noticeable that these obstacle-crossing parameters are predetermined by the experimenters.

**Figure 7:**
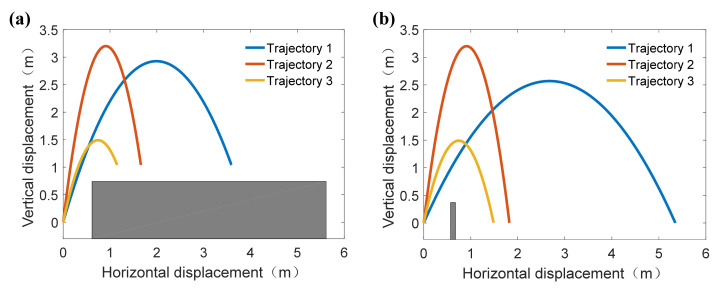
The optimal jumping trajectory under different weight coefficient combinations. (**a**) The optimal centroid jumping trajectories of jumping robot while jumping onto platform. (**b**) The optimal jumping centroid trajectories of jumping robot while jumping over the platform.

Therefore, the searching space was firstly determined by the decision criteria (3) and (5). Then the weight coefficient combinations were determined and the fitness function (29) was optimized by the MWOA. Lastly, the optimization results were listed in Table 5, where optimization I and II were for jumping onto a platform and jumping over a platform, respectively. The corresponding centroid jumping trajectories were shown in Figure 7.

**Figure 8:**
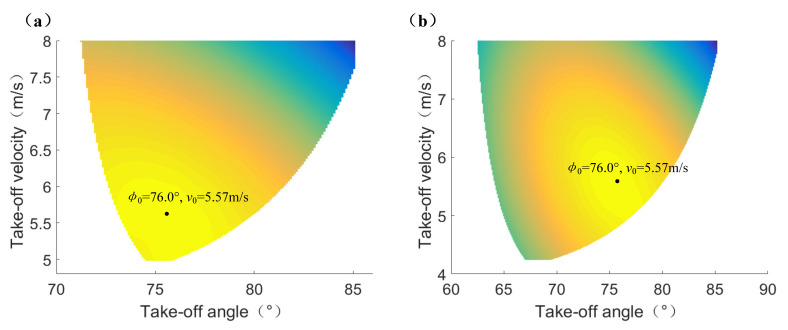
The optimization results of theoretical jumping trajectory. (**a**) The optimum take-off parameters for jumping onto the platform. (**b**) The optimum take-off parameters for jumping over the platform.

Figure 8a,b show the optimum take-off parameters under the third weight coefficient combinations in Table 5 for jumping onto the platform and jumping over the platform, respectively. It can be seen that the optimum take-off parameters for jumping onto the platform and jumping over the platform are both *φ*_0_ = 76.0°, *v*_0_ = 5.57 m/s in their separate searching spaces, thus the reasonability of optimization results of theoretical jumping trajectory can be verified.

### 3.2. Aerial Pitch Control Experiments

**Figure 9:**
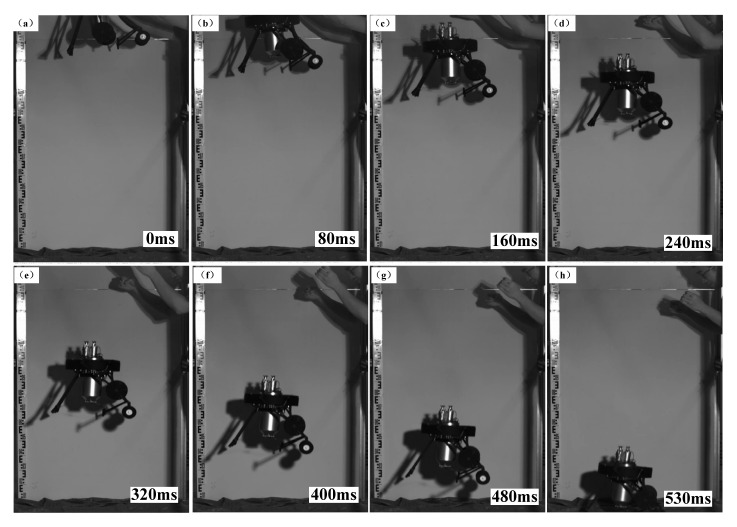
The time sequence of aerial pitch control experiment No. 4, Group 1.

**Figure 10:**
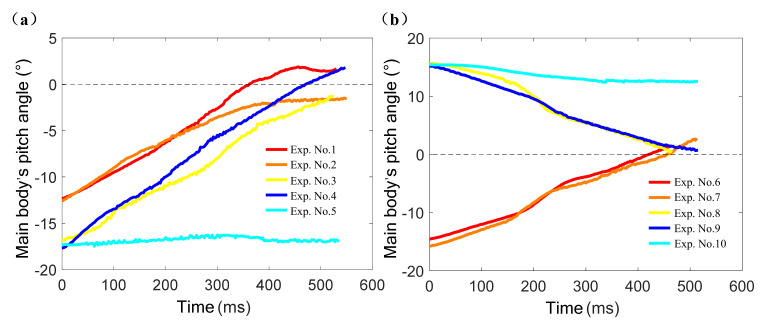
The jumping robot’s pitch angle curves of aerial pitch control experiment. (**a**) The pitch angle curves of Group 1. (**b**) The pitch angle curves of Group 2.

Figure 9 is a representative time sequence of aerial pitch control experiments (Experiment No. 4, Group 1). Figure 10 shows the jumping robot’s pitch angle curves of the aerial pitch control experiment, each curve started at the moment when the robot began to fall and ended at the moment when it contacted the ground. It can be seen from Figure 9 that the attitude sensor module detected the robot’s acceleration variation when the robot started falling freely, thus the assistant legs were driven to rotate a theoretical angle Δ*θ*_t_ = −48.5°. During the rotation of the assistant legs, the main body’s pitch angle also increased from the initial value −17.3°. According to Figure 10a, the robot’s pitch angle reached 0° at 422 ms and then 1.81° at 531 ms when the robot contacted the ground. After landing, the assistant legs reset to their initial position (parallel to the central axis of the water jet thruster), and the aerial pitch control process was completed.

As the expected value of pitch angle is 0°, it can be known from Figure 10 that the absolute values of maximum error of pitch angle in Group 1 and Group 2 were 1.81° (Experiment No. 4) and 2.79° (Experiment No. 6) respectively. However, the pitch angle did not change obviously in Experiment No. 5 and Experiment No. 10 which did not conduct aerial pitch control movements.

The RMSE (Root Mean Square Error) [34] of the pitch angle curves of Experiment No. 4, Experiment No. 5, Experiment No. 9, and Experiment No. 10 can be compared respectively to verify the effectiveness of aerial pitch control movement. Hence the calculation results of RMSE for Experiment No. 4, No. 5, No. 9, and No. 10 were: RMSE_4_ = 9.227°, RMSE_5_ = 16.821°, RMSE_9_ = 8.637°, RMSE_10_ = 13.379°, respectively. If it is obvious that RMSE_4_ < RMSE_5_ and RMSE_9_ < RMSE_10_, then the effectiveness of the mathematical model of aerial pitch control movement can be verified and it can be applied in the actual jumping and obstacle-crossing experiment for the jumping robot.

### 3.3. Jumping Experiments of the Robot

#### 3.3.1. Trajectory Accuracy Verification Experiments

**Figure 11:**
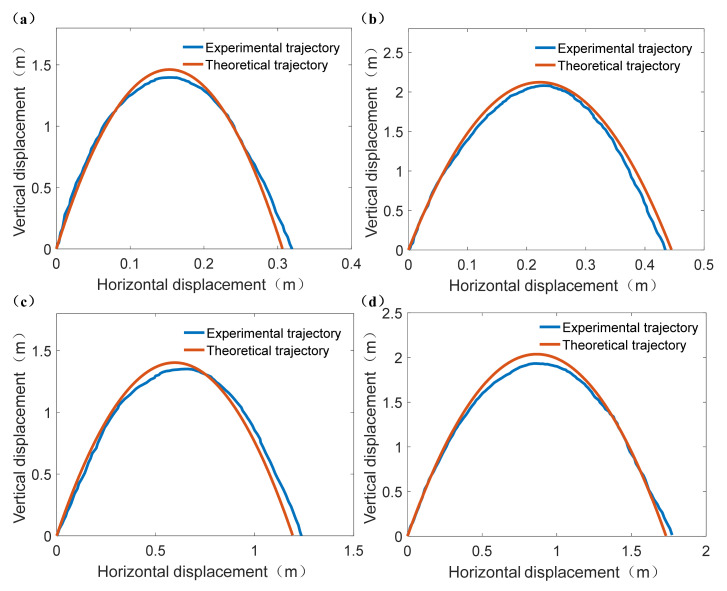
The comparisons of experimental jumping trajectories and theoretical jumping trajectories under different take-off parameters. (**a**) *φ*_0_ = 87°, *P*_max_ = 1.81 MPa. (**b**) *φ*_0_ = 87°, *P*_max_ = 2.23 MPa. (**c**) *φ*_0_ = 78°, *P*_max_ = 1.81 MPa. (**d**) *φ*_0_ = 78°, *P*_max_ = 2.23 MPa.

After the outdoor terrestrial jumping experiment, the four experimental jumping trajectories can be obtained and their comparisons with corresponding theoretical jumping trajectories were shown in Figure 11. Therefore, the RMSE of each experimental jumping trajectory can be calculated to evaluate its accuracy. The calculation results of RMSE for Figure 11a–d were: RMSE_a_ = 0.057 m, RMSE_b_ = 0.095 m, RMSE_c_ = 0.074, RMSE_d_ = 0.071 m, respectively. The maximum average degree of deviation in vertical displacement was 0.095 m, thus it can be concluded that the error between experimental jumping trajectory and theoretical jumping trajectory was small.

#### 3.3.2. Obstacle-Crossing Experiments

**Figure 12:**
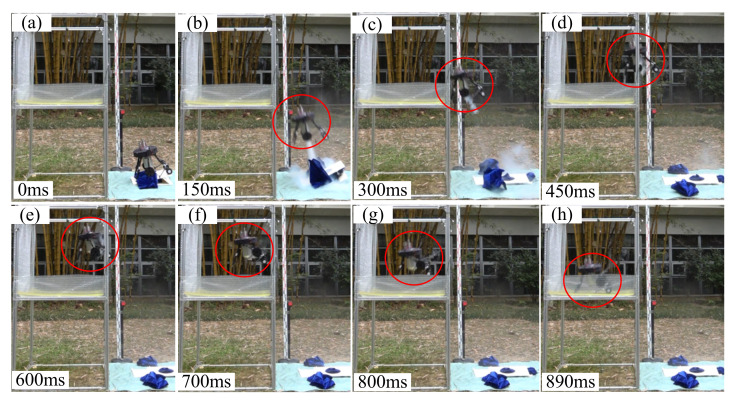
The time sequence of the jumping robot’s process of jumping onto the platform.

**Figure 13:**
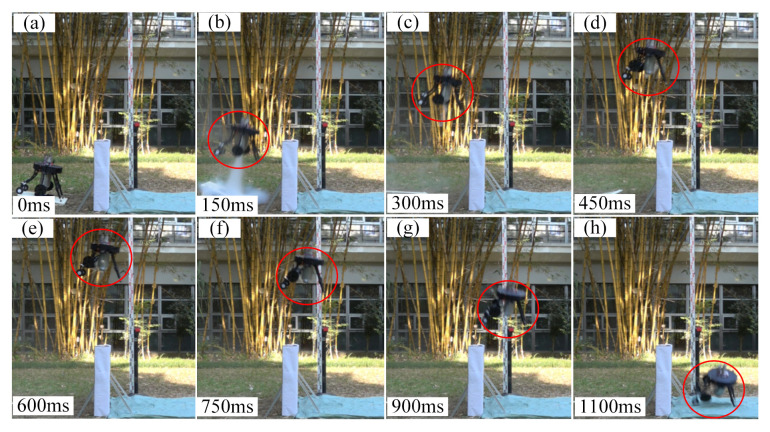
The time sequence of jumping robot’s process of jumping over platform.

**Figure 14:**
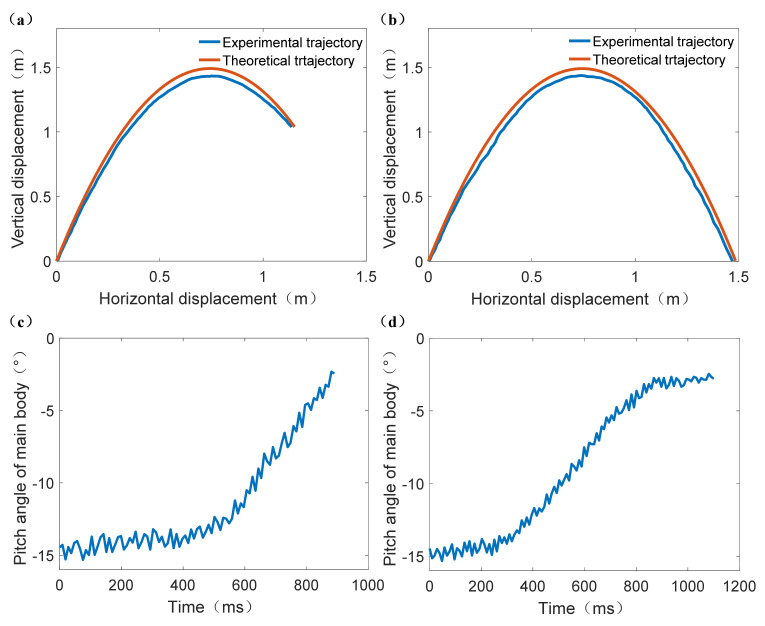
The jumping robot’s jumping trajectories and pitch angle curves of the obstacle-crossing experiment. (**a**) The comparison of jumping robot’s centroid trajectory of the experiment of jumping onto the platform. (**b**) The comparison of the jumping robot’s centroid trajectory of the experiment of jumping over the platform. (**c**) The pitch angle curve of the experiment of jumping onto platform. (**d**) The pitch angle curve of the experiment of jumping over platform. Both the desired pitch angle of main body in (**c**, **d**) are 0°.

The time sequence of jumping robot’s process of jumping onto platform and jumping over platform are shown in Figure 12 and Figure 13 respectively (The whole obstacle-crossing jumping processes are presented in https://www.youtube.com/watch?v=op6UnGVcYnw, (accessed on 30 March 2021) and in Video S1 (Supplementary Materials)). It can be known that the robot did successfully jump over and jump onto the platform with given take-off parameters. Although the robot did not compensate well in its roll direction due to the lack of attitude control action in the roll direction, the assistant legs’ rotation did have an obvious adjusting effect on the robot main body’s pitch angle. Then the jumping robot’s jumping trajectory comparisons and pitch angle curves of the main body are shown in Figure 14.

The comparison of the jumping robot’s centroid trajectory of the experiment of jumping onto the platform and jumping over the platform are shown in Figure 14a,b. It can be calculated that the RMSE values of these two experiments are RMSE_a_ = 0.0537 m and RMSE_b_ = 0.0731 m, respectively, which are both within the reasonable range. Thus, the accuracies of the robot’s jumping trajectory are acceptable.

According to Figure 14c, the robot’s pitch angle was stable at around −14° in the initial stage. Then the main body’s pitch angle started approaching the expected value of 0° in the effect of the assistant legs’ rotation after *t* = 550 ms. Lastly, the main body’s pitch angle increased to −2.45° at *t* = 890 ms when the robot landed on the platform. Thus, the duration and the absolute value of error of aerial pitch control was 340 ms and 2.45°, respectively. Similarly, in Figure 14d, the robot’s pitch angle was also stable at around −14° initially. Then the main body’s pitch angle started approaching the expected value of 0° after *t* = 290 ms. However, the pitch angle did not change obviously after *t* = 820 ms, and the final pitch angle was −2.79° at *t* = 1100 ms when the robot landed on the platform. Thus, the duration and the absolute value of error of aerial pitch control was 530 ms and 2.79° respectively.

Additionally, it can be calculated from Figure 14a that the initial experimental take-off angle and take-off velocity were *φ*_0e1_ = 75.54° and *v*_0e1_ = 5.15 m/s respectively for the experiment of jumping onto the platform, and therefore the corresponding fitness value was *f*_e1_ = 0.8328. Similarly, the calculation results for the experiment of jumping over the platform were *φ*_0e2_ = 75.53° and *v*_0e2_ = 5.24 m/s, respectively, therefore the corresponding fitness value was *f*_e2_ = 0.8337. The absolute value of relative error of fitness value for the experiment of jumping onto the platform and jumping over the platform were *e*_f1_ = 0.23%, *e*_f2_ = 0.12%. Therefore, the aerial pitch control model’s reasonability and the jumping robot’s accurate obstacle-crossing ability can be verified by these two obstacle-crossing experiments.

## 4. Conclusions

In this paper, a novel jumping robot was devised and the process of how it completed actual obstacle-crossing tasks and aerial pitch control movements are presented. Firstly, the decision criteria for overcoming general obstacles were proposed to obtain the feasible jumping trajectory set for the jumping robot. In order to optimize the jumping trajectory according to the fitness function value, a modified whale optimization algorithm (MWOA) was put forward and its performance superiority was verified by comparing it with other different intelligent optimization algorithms. Then the dynamics model of aerial pitch control was established and the jumping robot’s aerial pitch control experiment was conducted. The experimental results demonstrated the effectiveness of the proposed dynamics model. In addition, the robot’s trajectory accuracy verification experiment showed that the robot’s jumping trajectory was accurate and reliable. Finally, the robot’s obstacle-crossing experiments were conducted and the experimental results verified the robot’s good ability of obstacle-crossing and aerial pitch control. Collectively, these achievements provide meaningful guidance for the jumping robot’s performance in dealing with complex obstacle-crossing tasks and future research on jumping robot’s reliability as well as environmental adaptability.

## Figures and Tables

**Table 1:** Main components of jumping robot.

Component	Mass (g)	Amount
Water jet thruster	1348	1
Shell	1231	1
Front legs and dampers	485	2
Hind legs, driven wheels and payload	456	2
Servos of Assistant legs	121	2
Assistant legs	182	2
Driving wheels and their servos	256	2
Battery	138	1
Control electronics	147	1
Total	4364	-

**Table 2:** The experimental scheme of aerial pitch control of jumping robot.

	Experiment No.	Pitch Angle (°)	Initial Falling Height (m)	Assistant Leg’s Theoretical Rotation Angle (°)
Group 1	1	−12	1.35	−34.2
	2	−12	1.4	−34.2
	3	−17	1.35	−48.5
	4	−17	1.4	−48.5
	5	−17	1.4	/
Group 2	6	−15	1.05	−49.3
	7	−15	1.3	−49.3
	8	15	1.05	49.3
	9	15	1.3	49.3
	10	15	1.3	/

**Table 3:** Comparison of performance improvement effect of each improvement strategy.

Benchmark Function	Evaluation Indicator	MWOA-I	MWOA-II	MWOA-III	MWOA
*f*_1_	Mean value	1.05 × 10^−73^	**0**	1.54 × 10^−174^	**0**
	Standard deviation	4.82 × 10^−73^	**0**	0	**0**
*f*_2_	Mean value	1.59 × 10^−51^	**6.03 × 10^−210^**	4.19 × 10^−134^	1.27 × 10^−139^
	Standard deviation	6.30 × 10^−51^	**0**	2.92 × 10^−133^	6.94 × 10^−139^
*f*_3_	Mean value	5.64 × 10^4^	**0**	8.36 × 10^−176^	**0**
	Standard deviation	1.32 × 10^4^	**0**	0	**0**
*f*_4_	Mean value	2.80 × 10^1^	2.83 × 10^1^	2.86 × 10^1^	**2.77 × 10^1^**
	Standard deviation	4.17 × 10^−1^	3.20 × 10^−1^	2.60 × 10^−1^	**2.06 × 10^−1^**
*f*_5_	Mean value	−1.05 × 10^4^	−1.22 × 10^4^	**−1.25 × 10^4^**	−1.24 × 10^4^
	Standard deviation	1.74 × 10^3^	1.04 × 10^3^	**1.18 × 10^2^**	3.03 × 10^2^
*f*_6_	Mean value	1.89 × 10^−15^	**0**	**0**	**0**
	Standard deviation	1.04 × 10^−14^	**0**	**0**	**0**
*f*_7_	Mean value	3.97 × 10^−15^	**8.88 × 10^−16^**	**8.88 × 10^−16^**	**8.88 × 10^−16^**
	Standard deviation	2.59 × 10^−15^	**1.00 × 10^−31^**	**1.00 × 10^−31^**	**1.00 × 10^−31^**
*f*_8_	Mean value	3.66 × 10^−2^	**0**	**0**	**0**
	Standard deviation	9.24 × 10^−2^	**0**	**0**	**0**
*f*_9_	Mean value	2.22	3.29	1.75	**1.76**
	Standard deviation	2.02	3.57	1.87	**8.52 × 10^−1^**
*f*_10_	Mean value	7.12 × 10^−4^	6.52 × 10^−4^	3.98 × 10^−4^	**3.72 × 10^−4^**
	Standard deviation	4.75 × 10^−4^	1.83 × 10^−4^	1.09 × 10^−4^	**1.04 × 10^−4^**

**Table 4:** Performance comparison of MWOA with other intelligent optimization algorithms.

Benchmark Function	Evaluation Indicator	WOA	DA	HHO	MWOA
*f*_1_	Mean value	3.98 × 10^−73^	2.03 × 10^3^	4.57 × 10^−93^	**0**
	Standard deviation	1.97 × 10^−72^	1.10 × 10^3^	2.50 × 10^−92^	**0**
*f*_2_	Mean value	1.65 × 10^−50^	1.44 × 10^1^	2.07 × 10^−50^	**1.27 × 10^−139^**
	Standard deviation	7.67 × 10^−50^	5.58	1.09 × 10^−49^	**6.94 × 10^−139^**
*f*_3_	Mean value	4.16 × 10^4^	1.42 × 10^4^	1.88 × 10^−70^	**0**
	Standard deviation	1.09 × 10^4^	8.49 × 10^3^	8.12 × 10^−70^	**0**
*f*_4_	Mean value	2.79 × 10^1^	4.25 × 10^5^	**1.32 × 10^−2^**	2.77 × 10^1^
	Standard deviation	3.35 × 10^−1^	3.93 × 10^5^	**1.92 × 10^−2^**	2.06 × 10^−1^
*f*_5_	Mean value	−9.83 × 10^3^	−5.36 × 10^3^	**−1.26 × 10^4^**	−1.24 × 10^4^
	Standard deviation	1.92 × 10^3^	6.57 × 10^2^	**8.14 × 10^1^**	3.03 × 10^2^
*f*_6_	Mean value	3.31	1.88 × 10^2^	**0**	**0**
	Standard deviation	1.82 × 10^1^	4.36 × 10^1^	**0**	**0**
*f*_7_	Mean value	4.45 × 10^−15^	1.05 × 10^1^	**8.88 × 10^−16^**	**8.88 × 10^−16^**
	Standard deviation	2.09 × 10^−15^	1.47	**1.00 × 10^−31^**	**1.00 × 10^−31^**
*f*_8_	Mean value	3.70 × 10^−18^	1.86 × 10^1^	**0**	**0**
	Standard deviation	2.03 × 10^−17^	1.15 × 10^1^	**0**	**0**
*f*_9_	Mean value	2.50	**1.23**	1.29	1.76
	Standard deviation	2.91	**4.28 × 10^−1^**	9.75 × 10^−1^	8.52 × 10^−1^
*f*_10_	Mean value	5.96 × 10^−4^	2.24 × 10^−3^	3.78 × 10^−4^	**3.72 × 10^−4^**
	Standard deviation	2.98 × 10^−4^	3.42 × 10^−3^	2.27 × 10^−4^	**1.04 × 10^−4^**

**Table 5:** Optimization results of jumping trajectory under different weight coefficient combinations.

No.	Priority	Weight Coefficient Combination	Optimization Result I	Optimization Result II
1	Horizontal jumping distance	*ω*_1_ = 0.6132, *ω*_2_ = 0.2125, *ω*_3_ = 0.1743	*φ*_0_ = 71.2°, *v*_0_ = 8 m/s	*φ*_0_ = 62.5°, *v*_0_ = 8 m/s
2	Vertical jumping distance	*ω*_1_ = 0.0315, *ω*_2_ = 0.8149, *ω*_3_ = 0.1536	*φ*_0_ = 81.9°, *v*_0_ = 8 m/s	*φ*_0_ = 81.9°, *v*_0_ = 8 m/s
3	Landing safety	*ω*_1_ = 0.1852, *ω*_2_ = 0.0926, *ω*_3_ = 0.7222	*φ*_0_ = 76.0°, *v*_0_ = 5.57 m/s	*φ*_0_ = 76.0°, *v*_0_ = 5.57 m/s

## Data Availability

Not applicable.

## Supplementary Materials

The following are available online at https://www.youtube.com/watch?v=op6UnGVcYnw, Video S1: HITSZ_Jumping robot (accessed on 30 March 2021) and at https://www.mdpi.com/article/10.3390/s21072432/s1, Video S1: HITSZ_Jumping robot.

## Appendix A

**Table A1:** The benchmark functions of algorithm.

Name	Formula	Searching Range	Dimension	Minimum Value
Sphere	$f_{1}(x)=\sum_{i=1}^{\dim}x_{i}^{2}$	[−100,100]	30	0
Schwefel 2.22	$f_{2}(x)=\sum_{i=1}^{\dim}|x_{i}|+\prod_{i=1}^{\dim}\left|x_{i}^{}\right|$	[−10,10]	30	0
Schwefel 1.2	$f_{3}(x)={\sum_{i=1}^{\dim}\left(\sum_{j=1}^{i}x_{j}\right)}^{2}$	[−100,100]	30	0
Rosenbrock	$f_{4}(x)=\sum_{i=1}^{\dim-1}\left[100{\left(x_{i+1}-x_{i}^{2}\right)}^{2}+{\left(x_{i}-1\right)}^{2}\right]$	[−30,30]	30	0
Schwefel 2.26	$f_{5}(x)=\sum_{i=1}^{\dim}-x_{i}\sin(\sqrt{\left|x_{i}\right|})$	[−500,500]	30	−12,569.5
Rastrigin	$f_{6}(x)=\sum_{i=1}^{\dim}\left[x_{i}^{2}-10\cos(2\pi x_{i})+10\right]$	[−5.12,5.12]	30	0
Ackley	$\begin{array}{l} f_{7}(x)=-20\exp\left(-0.2\sqrt{\frac{1}{\dim}\sum_{i=1}^{\dim}x_{i}^{2}}\right)\\ -\exp(\frac{1}{\dim}\sum_{i=1}^{\dim}\cos(2\pi x_{i}))+20+e \end{array}$	[−32,32]	30	0
Griewank	$f_{8}(x)=\frac{1}{4000}\sum_{i=1}^{\dim}x_{i}^{2}-\prod_{i=1}^{n}\cos(\frac{x_{i}}{\sqrt{i}})+1$	[−600,600]	30	0
Shekel	$f_{9}(x)=[\frac{1}{500}+\sum_{j=1}^{25}\frac{1}{j+\sum_{i=1}^{2}{\left(x_{i}-a_{ij}\right)}^{6}}{]}^{-1}$	[−65,65]	2	1
Kowalik	$f_{10}(x)={\sum_{i=1}^{11}\left[a_{i}-\frac{x_{1}(b_{i}^{2}+b_{i}x_{2})}{b_{i}^{2}+b_{i}x_{3}+x_{4}}\right]}^{2}$	[−5,5]	4	0.00030
